# Genome and Epigenome Surveillance Processes Underlying UV Exposure in Plants

**DOI:** 10.3390/genes8110316

**Published:** 2017-11-09

**Authors:** Jean Molinier

**Affiliations:** Institut de Biologie Moléculaire des Plantes, UPR2357-CNRS, 12 rue du Général Zimmer, 67000 Strasbourg, France; jean.molinier@ibmp-cnrs.unistra.fr; Tel.: +33-03-67-15-53-41

**Keywords:** ultraviolet, photolesions, DNA repair, genome

## Abstract

Land plants and other photosynthetic organisms (algae, bacteria) use the beneficial effect of sunlight as a source of energy for the photosynthesis and as a major source of information from the environment. However, the ultraviolet component of sunlight also produces several types of damage, which can affect cellular and integrity, interfering with growth and development. In order to reduce the deleterious effects of UV, photosynthetic organisms combine physiological adaptation and several types of DNA repair pathways to avoid dramatic changes in the structure. Therefore, plants may have obtained an evolutionary benefit from combining genome and surveillance processes, to efficiently deal with the deleterious effects of UV radiation. This review will present the different mechanisms activated upon UV exposure that contribute to maintain genome and integrity.

## 1. Introduction

The sun emits radiation that reaches the Earth's surface. Ultraviolet (UV; 10–400 nm), visible light (390–750 nm), and infrared (IR; 750–10^6^ nm) are part of this radiation that contribute, to different extents, to plant development. Visible light, more specifically blue and red light, is absorbed for photosynthesis [1]. Near IR (far-red) participates in the activation of different developmental programs, such as the de-etiolation and the induction of flowering [2]. However, an excess of IR leads to enhanced temperature (heat stress) requiring adaptation, avoidance, and acclimation processes [3]. 

The harmful UV-C radiation (100–280 nm) emitted by the sun is absorbed by ozone and dioxygen [4]. Therefore, only UV-A (95% of the total UV) and UV-B (5%) reach the Earth’s surface and, therefore, are biologically relevant. Indeed, UV-A/blue light and UV-B photoreceptors trigger numerous transcriptional changes and nuclear reshapings, leading to the efficient induction and control of developmental programs [2,5,6]. On the other hand, UV radiation has damaging effects on the cells and the genome [4]. In order to reduce these deleterious effects, photosynthetic organisms produce UV-absorbing phenolic compounds, i.e., flavonoids, acting as sunscreen pigments [7], and modify their cuticular waxes composition to enhance reflectivity and act as a photoprotective layer [8]. Upon UV exposure, genes encoding for key enzymes involved in secondary metabolites synthesis, as well as for antioxidant factors are transcriptionally activated [9]. Additionally, rapid nuclei movements are established to counteract the genotoxic effect of UV [10]. Ultraviolet induces DNA photolesions directly, and base modifications indirectly, the latter through oxidation that could interfere with DNA replication and transcription [11]. The UV-induced DNA damage must be efficiently repaired in order to prevent mutations or improper chromosome rearrangements that could be transmitted to the progeny [12]. Direct repair (DR), also called photo reactivation, and nucleotide excision repair (NER) are the main pathways used to repair DNA photolesions [13]. Additionally, base excision repair (BER) and double-strand break repair (DSB) are activated to process base modifications or repair intermediates that are indirectly produced by UV irradiation [12]. Interestingly, plants deficient in the expression of factors involved in the regulation of chromatin structure also exhibit genotoxic stress hypersensitivity [14,15,16], suggesting that the processes maintaining genome and epigenome integrity are closely linked. 

In this review, we will describe the impact of UV exposure on genome and epigenome structures as well as the DNA repair mechanisms activated by UV. Moreover, we will review the strategies that plants activate to coordinate or to interconnect the different processes that contribute to the maintenance of epigenome integrity.

## 2. Ultraviolet-Induced DNA Damage

Both UV-B and UV-C are absorbed by DNA bases and thus directly produce photolesions [4]. Cyclobutane pyrimidine dimers (CPDs) involving thymine (T) and cytosine (C) and occurring in TT, CC, TC, and CT contexts, and 6–4-photoproducts (6–4 PP) are the main types of UV-induced DNA damage [4]. These photoproducts lead to DNA helix distortion, interfering with DNA replication and transcription. To a lower extent, UV-B and UV-C indirectly oxidize DNA bases via the production of reactive oxygen species (ROS) that predominantly modify guanine (G), giving rise to 8-oxo-7,8-dehydroguanine (8-oxoG) [4]. 

Differently from UV-B/C, UV-A is poorly absorbed by the DNA, and thus photoproducts are moderately generated [4]. As a result of a massive production of ROS upon UV-A exposure, 8-oxoG and DNA single-strand breaks (SSBs) are formed. DNA double-strand breaks (DSBs) are also indirectly produced, likely because of the generation of intermediates during the activation of DNA repair processes [17]. In addition to the aforementioned DNA damage, UV irradiation could lead to more complex lesions, such as tandem base damage as well as DNA–DNA and DNA–protein crosslinks [18].

Interestingly, the amount of UV-B-induced DNA damage, UV sunscreen pigments, and the efficiency of light-dependent repair differ between dicot, monocot, herbaceous, and woody species, highlighting that UV-B tolerance mechanisms and strategies are differentially balanced between species [19]. 

Finally, the mapping of UV-induced DNA damage in humans (CPDs and 6–4 PPs) and in plants (CPDs) showed that photolesions are randomly and uniformly distributed throughout the genome, albeit pyrimidine richness and combination likely determine DNA responsiveness, including the rate of excision and the repair efficiency [20,21,22].

## 3. Ultraviolet-Induced Genomic Changes

Ultraviolet radiation indirectly modifies genome sequence and structure. In cytosine-containing photoproducts (CC, CT, and TC) nucleotide transitions (G:C–adenine (A):T) are predominantly produced [23,24], while changes from CC to TT have been reported to a lower extent [23]. Interestingly, genes were more prone to nucleotide transitions under UV-B conditions than transposable elements (TEs) [24]. The oxidatively induced DNA modification (8-oxoG) leads to a mismatch with adenine resulting in G to T and C to A substitutions [25].

At a larger genomic scale, chromosome rearrangements are induced by photosynthetically active radiation, as measured using somatic homologous recombination (HR) reporter constructs [26,27]. This holds true upon UV-B and UV-C exposure [28,29], strengthening that high light/irradiation leads to genomic changes. The mobilization of TEs also induces genome rearrangements called insertions/deletions (InDel). Interestingly, it has been reported that in maize the *Mu* TE is reactivated by UV-B treatments [30], and new insertions events could be detected [31]. 

## 4. Ultraviolet-Induced Epigenome Dynamics

Epigenome changes are associated with transcriptional activation, repression, or both as well as with chromatin remodelling. UV-B and UV-C deregulate the expression of hundreds of genes [32,33]. Light signaling integrators, such as de-etiolated 1 (DET1) and constitutive photomorphogenic 1 (COP1), control the level of heterochromatin decondensation in etiolated tissues, while photoreceptors trigger heterochromatin compaction under light conditions [5]. This emphasizes that light-dependent mechanisms modulate, globally or specifically, chromatin structure to trigger genome and epigenome structuration. 

Epigenetic marks, such as histones post-translational modifications (PTM), actively participate in the regulation of light-dependent processes. The histone lysine (K) demethylase H3K36 (KDM4) regulates the expression of photolyase 1 *(PHR1)* that is involved in the repair of UV-induced DNA lesions in a light-dependent manner [34]. In maize, UV-B induces the maintenance of histone repressive marks at the terminal inverted repeats (TIRs) of the TE *Mu* [35]. Moreover, the binding of the *Mu* transposase (MURA) is enhanced within *Mu DR* TIRs upon UV-B exposure, suggesting that its mobilization is regulated by UV stress [31]. 

The chromatin environment also participates in the regulation of the DNA damage response. The chromatin state in the vicinity of a damaged site undergoes rapid remodeling to signal and allow access to the DNA repair factors [36]. In mammals and in plants, phosphorylation of the histone H2A.X (γH2A.X) triggers the recruitment of DNA repair proteins at DSBs and activates checkpoint proteins which arrest the cell cycle progression [37,38]. Additionally, increased levels of γH2A.X could also be detected in mitotic cells in the absence of DNA damage [39].

Phosphorylation of another histone variant, H2A.W.7, was reported to be involved in the DNA damage response in highly condensed heterochromatin, whilst γH2A.X was primarily detected in euchromatin [40]. 

In response to DNA damage, chromatin reconstruction plays an important role. The histone chaperones nucleosome assembly protein 1 (NAP1) and chromatin assembly factor-1 (CAF-1) regulate the expression of genes involved in DNA repair as well in nucleosome disassembly and reassembly during NER and HR [41,42,43]. 

Another important epigenetic mark is DNA methylation, i.e., 5-methyl cytosine (5-mC). DNA methylation patterns are the dynamic outcome of methylation and demethylation processes that are required for the stable silencing of TEs as well as for the regulation of gene activity [44]. In response to UV-B radiation, a loss of DNA methylation was measured in *Artemisia annua* at seven putative transcription factor binding sites [45]. In maize, minimal changes in DNA methylation were observed upon UV-A/B irradiation [46].

Genome–epigenome structure and organization also determine how DNA is damaged and how UV responsiveness is triggered. It has been reported that 5-mC adjacent to pyrimidine has higher absorbance in the UV-B range, and is therefore more prone to form pyrimidine dimers compared to unmethylated cytosines [47]. Since DNA methylation is concentrated to specific genomic regions, this may suggest that regions enriched in 5-mC, e.g., constitutive heterochromatin, may represent hot spots of photodamage and may influence genome stability and flexibility. In agreement with this observation, it was demonstrated a strong positive correlation between DNA methylation patterns and mutations induced by UV irradiation in *Arabidopsis thaliana* [24]. 

## 5. Repair of UV-Induced DNA Damage

### 5.1. Direct Repair

**Figure 1:**
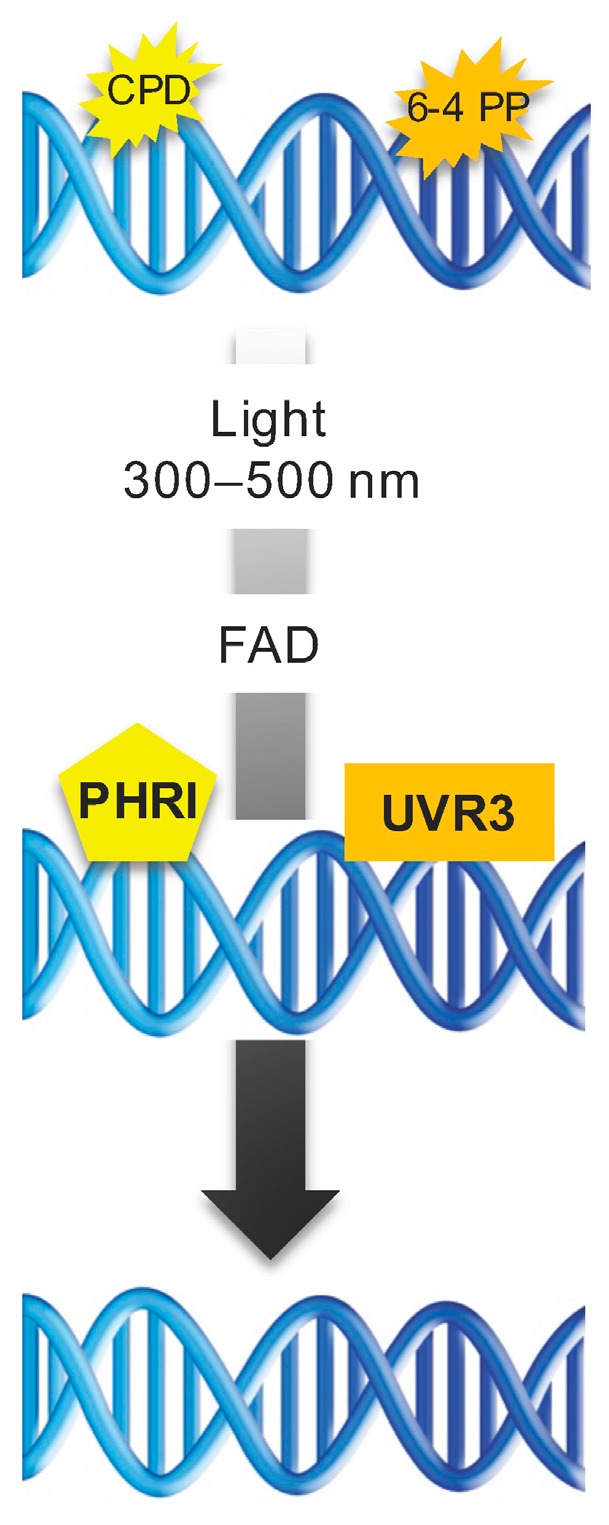
Direct repair (DR) pathway. Schematic representation of the light-dependent repair pathway of cyclobutane pyrimidine dimer (CPD)- and 6–4-photoproducts (6–4 PP)-induced DNA damage.

Ultraviolet-induced DNA lesions are preferentially repaired by DR using photolyases. DR is an error-free mechanism of repair [48]. Prokaryotes and most eukaryotes, excepting placental mammals, predominantly use this direct mechanism to repair DNA photolesions [48]. Photo reactivation is catalyzed by specific enzymes called DNA photolyases, which directly bind UV photoproducts and convert pyrimidine dimers to monomers using the cofactor flavine adenine dinucleotide (FAD) and UV-A/blue light (300–500 nm) [48] (Figure 1). In *A. thaliana*, two active photolyases are found: PHR1, also named UV resistance 2 (UVR2), acting specifically on CPDs, and UV resistance 3 (UVR3) acting specifically on 6,4 PPs [11] (Figure 1). Both photolyases prevent the accumulation of UV-B-induced genetic defects [24,28]. Moreover, the expression of these photolyases UVR3 and PHRI is controlled by the photomorphogenic regulator DET1 [49] and the DNA demethylase repressor of silencing 1 (ROS1) [15], highlighting that an interplay between photomorphogenesis, DNA methylation dynamics, and light-dependent repair mechanisms exists. 

### 5.2. Nucleotide Excision Repair

**Figure 2:**
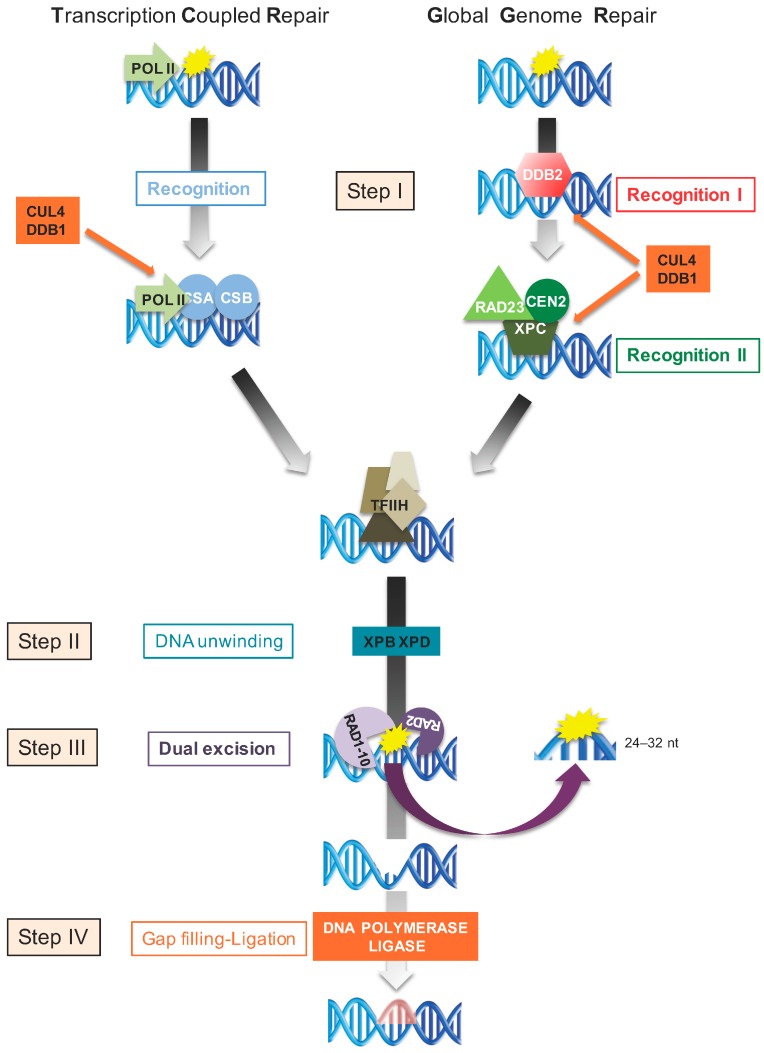
Nucleotide excision repair (NER) pathways. Schematic representation of the four main steps of the two NER subpathways: transcription-coupled repair (TCR) and global genome repair (GGR). The recognition of photolesions (Step I) is performed by TCR and GGR specific factors. One recognition complex acts during TCR, whereas two recognition complexes are necessary for GGR. Subsequently, for both TCR and GGR, DNA helicases open the chromatin (Step II), allowing the excision of the damaged DNA strand (Step III) and its synthesis-dependent repair (Step IV). Yellow star: photolesions. Newly synthetized DNA is shown in red.

Nucleotide excision repair promotes the repair of UV-induced lesions in a light-independent manner via two sub-pathways, the transcription-coupled repair (TCR) and the global genome repair (GGR), that process bulky DNA lesions along actively transcribed DNA strands or throughout the genome, respectively [50] (Figure 2). The NER process removes DNA photolesions by the excision of 24–32 nucleotides (nt) single-strand oligonucleotides from the damaged DNA strand, followed by the restoration of an intact double helix by DNA repair synthesis (Figure 2). About 30 core proteins are mobilized during this repair process [50]. Although the recognition steps differ between TCR and GGR, the excision repair processes are similar (Figure 2).

### 5.3. Transcription-Coupled Repair

In actively transcribed genomic regions, photoproducts block transcription. The stalled RNA polymerase II (RNA POL II) triggers the recognition signal that allows the recruitment of the cockayne syndrome protein A and B (CSA, CSB, Figure 2) [50]. These proteins help to change the interface between RNA POL II and the damaged sites [50]. Subsequently, CSA proteins are ubiquitinated by the ubiquitin E3-ligase complex DNA cullin-4-DNA damage-binding protein 1 (CUL4-DDB1) and degraded by the 26S proteasome [50] (Figure 2). Upon this recognition step, the multiprotein complex transcription factor II human (TFIIH) is recruited to the damage site [50] (Figure 2).

### 5.4. Global Genome Repair

In poorly transcribed and untranscribed DNA strands, GGR repairs DNA photolesions. The DNA damage-binding protein 2 (DDB2) is the main factor involved in the recognition of UV-induced DNA lesions [51,52] (Figure 2). The DDB2, through its DNA binding domain, senses photolesions, abasic sites, and G·T mismatches [53]. Bulky lesions removal entails a tight control of DDB2’s turnover by the ubiquitin E3-ligase complex CUL4-DDB1 and the DNA damage signaling kinase ataxia-telangiectasia RAD3-related (ATR; [52,54]). Subsequently, a second recognition complex, *xeroderma prigmentosum* complementation group C (XPC), together with radiation sensitive 23 (RAD23) and centrin 2 (CEN2), is mobilized at the damaged sites, and binds the strand opposite the lesion [55] (Figure 2). This helps to recruit the TFIIH complex [50]. Several studies have shown that unexpected factors also participate in GGR. Indeed, the repressor of photomorphogenesis DET1 cooperates with DDB2 during the excision repair process of DNA photolesions [56]. Moreover, it has been recently demonstrated that, upon UV irradiation, the post-transcriptional gene silencing (PTGS) factor argonaute 1 (AGO1), together with DDB2, forms a chromatin-bound complex likely recognizing the photolesions in an RNA–DNA complementary, strand-specific manner [22]. Interestingly, the biogenesis of these photoproduct-associated siRNAs involves transcriptional gene silencing (TGS) and PTGS factors, strengthening that a complex cross-talk between epigenomic and genomic maintenance pathways exist.

### 5.5. Dual Incision, Repair Synthesis and Ligation

Once the TFIIH complex is recruited to the repair site, both TCR and GGR pathways converge for the removal of the DNA photoproducts. Two ATPases/helicases, *xeroderma prigmentosum* complementation group B and D (XPB and XPD), unwind the DNA to create a 20–30 nt bubble (Figure 2). The endonucleases radiation sensitive 1 and 10 (RAD1-RAD10) and radiation sensitive 2 (RAD2) perform an incision about 20 nt 5’ and 5 nt 3’ to the lesion, respectively [50] (Figure 2). This dual incision allows the release from the genome of oligonucleotides of 24 to 32 nt in length [50] (Figure 2). The DNA polymerase (DNA POL), delta and/or epsilon in mammalian cells and yeast, fill the gap [57], and the DNA ligase I seals the DNA (Figure 2). In *A. thaliana* the DNA POL involved in the synthesis-dependent repair remains to be clearly characterized, although it is speculated that the DNA POLε could play this role [50].

### 5.6. Repair of Oxidatively Induced DNA Damage by the Base Excision Repair

**Figure 3:**
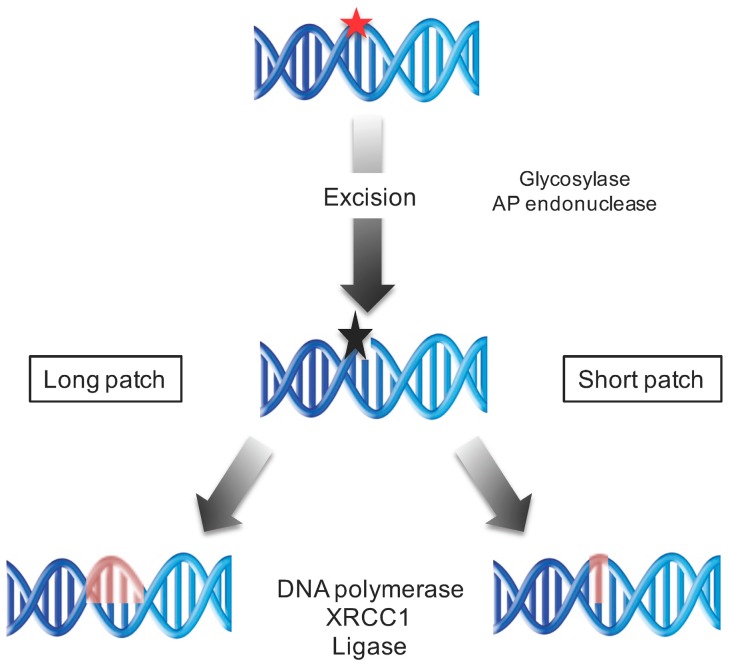
Schematic representation of the base excision repair (BER) pathway. The modified base is recognized and excised by specific DNA glycosyleases creating an abasic site. The gap is filled by a DNA polymerase and ligated by a DNA ligase and X-ray repair cross-complementing protein 1 (XRCC1). Red star: modified base; black star: 3’ blocking end. The newly synthetized DNA is shown in red.

8-Oxo-7,8-dehydroguanine is caused by the massive production of ROS generated upon UV irradiation [58]. The BER pathway (short and long patches; Figure 3) contributes to repair these oxidatively induced DNA lesions [13]. The mechanisms of BER are highly conserved from bacteria to humans. BER consists in recognizing the modified nucleobase and then cleaving the *N*-glycosyl bond to release the modified base, thus forming an abasic site. This process is performed by DNA glycosylases specific for the modified base. Two different types of DNA glycosylases exist. The monofunctional glycosylases remove the modified base, and then the abasic site is cleaved by an apurinic endonuclease (APE), whereas the bifunctional glycosylases possess both activities. In *A. thaliana* 8-oxoguanine DNA glycosylase 1 (OGG1) is involved in the BER of 8-oxoG [59]. In mammalian cells and yeast, the DNA POL β/δ/ε fills the single nucleotide gap, and the nick is sealed by the DNA ligase III/ X-ray repair cross-complementing protein 1 (XRCC1) [13] (Figure 3). In plants, the DNA POL involved in BER remains to be identified. 

Oxidatively induced DNA damage is indirectly induced upon UV exposure, therefore it cannot be excluded that other base modifications also appear (i.e., alkylation, deamination) that must, as well, be efficiently repaired by BER-related processes.

## 6. Double-Strand Break Repair

DNA DSBs are also indirectly generated by UV, even if t o a lower extent [11]. This harmful type of damage must be rapidly and efficiently repaired to avoid cell death. Two main processes repair DSBs: non-homologous repair (NHR) that frequently leads to insertions or deletions of DNA sequences, and HR that is stated as a more accurate pathway. In higher eukaryotes, the predominant DSB repair pathway is NHR [60]. It is important to note that both processes lead to variable size insertions and deletions (<3 bp) and to sequence rearrangements [61]. Interestingly, DR- and GGR-deficient plants exhibit higher somatic HR [28,29], emphasizing that interconnections between the DNA repair pathways exist. Moreover, it could be speculated that this enhancement of somatic HR could be likely due to a temporal extension of the unrepaired photoproducts or to the accumulation of unprocessed DNA repair intermediates.

## 7. Interplay between Genome and Epigenome Maintenance Processes

Surprisingly, several studies have uncovered that DNA repair factors control the shaping of the DNA methylation landscape. In *A. thaliana* a loss of function of the mismatch repair factor, MutS protein homolog 1 (MSH1), in plastids induces heritable alterations of the DNA methylation patterns through an unknown mechanism [62]. In *A. thaliana*, loss of the GGR factor, DDB2, alters the DNA methylation patterns at many repeated loci and protein-coding genes [63]. Indeed, DDB2 acts in a complex that includes argonaute 4 (AGO4) to control de novo DNA methylation via the modulation of the local abundance of 24 nt small interfering RNAs (siRNAs) [63]. On the other hand, decreased DNA methylation 1 (DDM1), a nucleosome remodeler involved in the maintenance of DNA methylation, and ROS1, a primary factor required for active DNA demethylation, have also been found to be involved in UV-B DNA damage repair [15]. Given that active DNA demethylation is a DNA repair-related mechanism, it could be guessed that DNA repair factors may, directly or indirectly, act in the control of DNA methylation dynamics. In mammals, NER has been found to contribute to active DNA demethylation at particular loci to regulate gene expression [64,65]. Interestingly, in *A. thaliana*, DDB2 negatively regulates the expression of *ROS1* [63], emphasizing that a complex interplay between DNA repair, and DNA methylation and demethylation pathways exists. Moreover, these studies allow speculating that the regulation of DNA methylation dynamics would likely be a *bona fide* response to UV exposure. Genome stability and plasticity are coregulated by complex phenomena. Indeed, it has been assumed that the combination of several different processes involved in the maintenance of genome integrity could explain the different strategies of genome size and karyotype evolution, such as the DSB repair pathways, and the whole-genome duplication (WGD) [66,67]. For example, genome dynamics, as measured by somatic HR frequency, are stimulated upon UV stress exposure and persist in subsequent, untreated generations of *A. thaliana* plants [29,68], suggesting that some epigenetic features, that still remain to be identified, have been established and maintained upon UV irradiation. Nevertheless, this transgenerational memory phenomenon is not a general response to abiotic stress [69]. Another aspect of genome dynamics, with a strong epigenetic component, is the stress-induced mobilization of TEs, which are thought to play a major role in genome evolution [70] . Indeed, various environmental stresses can release TE silencing [71,72,73,74,75], and TEs evolve *cis*-regulatory elements that may significantly contribute to stress adaptation [76].

There is emerging evidence that the outcomes of the genome rearrangements and of the repair strategies of damaged sites are influenced by the chromatin states. For example, in human cells, the active chromatin and inter-nucleosomal UV-damaged regions are repaired faster than the repressed (i.e., heterochromatic) and intra-nucleosomal regions [77,78]. Multiple chromatin assembly factors and chromatin remodeling complexes have also been found to be necessary for resistance against genotoxic stresses, for normal levels of HR, and for the maintenance of genome integrity [16,79,80,81]. These findings show that genome dynamics upon stress exposure, including DNA damage, are controlled by epigenetic factors and can possibly have effects on plant adaptation and evolution. It is tempting to speculate that genomic regions carrying particular chromatin states are more reactive to UV irradiation than other regions. For example, DNA methylation patterns correlate with the geography and the climate of origin, supporting the idea that this epigenetic mark plays a role in the adaptation to local environments that likely include the intensity of UV radiation [82]. More generally, a remarkable variation of DNA methylation was identified in angiosperms [83], showing that the DNA methylation landscapes reflect the evolutionary and life histories of plant species related to ecological niches [82]. Collectively, these findings allow proposing that genome dynamics, upon stress exposure, may be under the influence of epigenetic factors and can affect adaptation and evolution [84]. They also suggest that the ability of genomic regions to produce photolesions (i.e., heavily methylated loci), and the interplay between processes involved in the maintenance of genome and epigenome integrity exist. 

Finally, we have to consider that most of the pathways used to repair UV-induced DNA lesions (NER, short and long patch BER, and HR) are DNA synthesis-dependent repair processes. This implies that a new DNA strand is synthetized and that the associated epigenetic marks must be properly re-established in order to maintain the epigenomic landscape integrity (i.e., DNA methylation, histone post-translational modifications (PTMs)). Therefore, deciphering the chromatin-dependent mechanisms and their interplay involved in repairing and reshaping the plant epigenome upon DNA synthesis-dependent repair represent a key challenge. 

## 8. Conclusions and Outlook

The response and the adaptation to high light and UV stresses are a combination and a coordination of a large repertoire of responses ranging from physiological adaptation to genome and epigenome surveillance processes. The damaging effect of UV modifies DNA and chromatin structure triggering epigenome dynamics. However, the underlying molecular mechanisms need to be better characterized considering that a complex interplay between core DNA repair factors and epigenome surveillance systems exists. Deciphering how UV radiation directly and indirectly shapes the epigenome would help to understand how processes regulating stability and flexibility are balanced. Such studies would provide biomarkers (i.e., small RNA, damaged loci, etc.) to profile and anticipate the responses to UV-induced DNA damage.

In addition, UV stress rarely comes in the absence of heat stress. Thus, it would be very important to consider both stresses simultaneously. It is crucial to understand how plants deal with these multifactorial stresses to decipher the molecular mechanisms that are activated. In recent years, it has been shown that heat stress can have an important impact on the genome and epigenome of *A. thaliana* plants [71,72]. Therefore, it would be relevant to consider the interaction between UV and heat stresses, which are main factors influencing genome and epigenome stability.
